# Development of the Final Version of the Classification and Assessment of Occupational Dysfunction Scale

**DOI:** 10.1371/journal.pone.0134695

**Published:** 2015-08-11

**Authors:** Mutsumi Teraoka, Makoto Kyougoku

**Affiliations:** 1 Doctor Course, Graduate School of Health Sciences, Kibi International University, Okayama, Japan; 2 Oosugi Hospital, Okayama, Japan; 3 Department of Occupational Therapy, School of Health Sciences, Kibi International University, Okayama, Japan; Universität Bochum, GERMANY

## Abstract

Occupational therapy is involved in disability prevention and health enhancement through the prevention of occupational dysfunction. Although many occupational dysfunction scales exist, no standard method is available for the assessment and classification of occupational dysfunction, which may include occupational imbalance, occupational deprivation, occupational alienation, and occupational marginalization. The purpose of this study was to develop the final version of Classification and Assessment of Occupational Dysfunction (CAOD). Our study demonstrated the validity and reliability of CAOD in a group of undergraduate students. The CAOD scale includes 16 items and addresses the following 4 domains: occupational imbalance, occupational deprivation, occupational alienation, and occupational marginalization.

## Introduction

Occupational dysfunction is recognized worldwide as a major health-related problem in the field of preventive occupational therapy [1–3]. Occupation is considered to be the center of the human experience; it includes things people need to do, want to do, and are expected to do [4,5]. In particular, occupation includes many categories such as leisure, housework, sleep, and personal care [6]. Occupation not only involves work, business, and labor but also includes a wide range of activities such as education, play, activities of daily living, rest, and social participation.

Occupational dysfunction is defined as a negative experience related to engaging in daily activities, and it includes occupational marginalization, occupational imbalance, occupational alienation, and occupational deprivation [7]. Occupational dysfunction may present without obvious medical disease [8]. Moreover, risk of experiencing an occupational dysfunction is not confined to only adult workers but also to people in various developmental stages such as puberty, adolescence, and old age. Occupational marginalization is defined as a person not having the opportunity to engage in desired daily activities [9]. Occupational imbalance is defined as a loss of balance in engaging in daily activities [10]. Occupational alienation is defined as a situation when the inner needs of the individual related to daily activities are not satisfied [11]. Occupational deprivation is defined as a lack of opportunity for daily activities beyond the individual’s control [12]. These problems are barriers to social participation and lead to a decrease in health-related quality of life [13]. Preventive occupational therapy requires a valid and reliable assessment tool for occupational dysfunction.

Therefore, we developed a prototype assessment tool called the Classification and Assessment of Occupational Dysfunction (CAOD) [14,15]. The psychometric properties of the prototype were examined in 287 undergraduate students [14]. The rationale for sample choice is that undergraduate students are at high risk of occupational dysfunction because of an irregular lifestyle, poor sleep, dietary abnormalities, academic failure, and human relationship problems [16,17]. Analytical results supported a 4 factor model consisting of 19 items [14]. Overall, the validity and reliability of the prototype were satisfactory [14]. The CAOD prototype was based on the Occupational Based Practice 2.0 (OBP2.0) [14,15]. OBP2.0 was developed by the integration of theoretical study and clinical study in Japan [7]. Since then, various occupational therapists have been involved in its further development [18]. In Japan, OBP2.0 is utilized in a variety of circumstances. For example, OBP2.0 has been applied to diverse patient groups with acute and chronic orthopedic injuries, patients with mental illness, older persons with dementia, children with developmental disorders, and persons with cerebrovascular disorders. OBP2.0 is designed to help the people with occupational dysfunction related to occupational marginalization, occupational imbalance, occupational alienation, and occupational deprivation. OPB2.0 also includes a dissolution approach for belief conflict (DAB) for conflict management [7]. DAB was developed as a comprehensive intervention program for people who are suffering from belief conflicts [7,18–22]. Therefore, OBP2.0 is a viable solution for occupational dysfunction and clarification of belief conflict [7]. The prototype CAOD based on OBP2.0 is focused on the assessment and classification of occupational dysfunction [14,15].

The factor structure of the prototype CAOD was different from the theoretical expectation in several ways [14]. First, the prototype CAOD suggested occupational functioning as a new factor in addition to occupational marginalization, occupational imbalance, occupational alienation, and occupational deprivation [14]. This is surprising because occupational functioning is the opposite of occupational dysfunction [2,3]. Therefore, occupational functioning was operationally defined as an individual’s positive experiences related to engaging in daily activities [14]. Second, occupational alienation and occupational deprivation were integrated as a single factor by exploratory factor analysis (EFA) [14]. For these reasons, we reexamined the psychometric properties and item pools of the prototype CAOD for the development of a final version of CAOD [14].

The purpose of this study was to develop the final version of CAOD and examine its validity and reliability in a group of undergraduate students.

## Methods

### Ethics statement

The research protocol was approved by the Ethics Committee of Kibi International University (No. 11–22) and the Research Ethics Committee of Saitama Prefectural University (No. 23068). All participants provided written and verbal informed consent prior to participating. Participation was voluntary, and participants had the right to dropout from the research at any time without giving reason. This study was conducted according to the Declaration of Helsinki.

### Development of item pool

To develop the item pool of CAOD, we systematically identified extant items relevant to occupational functioning, occupational marginalization, occupational imbalance, occupational alienation, and occupational deprivation. Using these 5 concepts, we compiled an initial item pool of 40 items consisting of: (1) occupational functioning (8 items), (2) occupational marginalization (8 items), (3) occupational imbalance (8 items), (4) occupational alienation (8 items), and (5) occupational deprivation (8 items). The planned number of items for the final version CAOD was 10–15. Relevant assessments included the work-family conflict scale [23], the sense of isolation scale [24], the scale of the sense of fulfillment of life [25], the Beck depression inventory [26], and many other assessments.

To assess the instrument’s content and face validity, a panel of 4 occupational therapy experts (specialists in 2 physical areas and 2 psychosocial areas) reviewed the OBP2.0 item pool. We performed the expert consensus protocol in 3 steps: Step 1, item pool review; Step 2, item adjustment; and Step 3, final review. In Step 1, the item pool was reviewed to assess each question for content and face validity related to the 5 concepts. The content and face validity of 40 items were rated on a 2 point Likert scale, with 1 being “least plausible” and 2 being “most plausible.” For inclusion in the item pool of CAOD, an item needed the consent of 75% of the experts in the panel. If the Likert rating was 1, the panelist was asked to suggest additional examples, modifications of wording, or deletions, as appropriate. In Step 2, we revised the language of the collected questions to reflect the results of the Step 1. In Step 3, the panelists reviewed the 40 items again to ensure that the classification of occupational dysfunction adequately represented the 5 domains in the item pool. A total of 40 items of CAOD successfully passed through this process and were sent for field-testing.

### Participants

All participants were enrolled in the Kibi International University or the Saitama Prefectural University. A description of the research was given at the end of lectures. A total of 731 participants were contacted. These included 330 undergraduate occupational therapy students, 191 undergraduate physical therapy students, and 210 undergraduate nursing students.

### Measures

#### Participant profile

We obtained relevant demographic data from participants: gender, age, school year, and department.

#### 40 item version of Prototype CAOD

The 40 item version of the Prototype CAOD was used to assess occupational dysfunction types, including occupational marginalization, occupational imbalance, occupational alienation, occupational deprivation, and occupational functioning, based on OBP2.0. The CAOD item design was based on a 7 point Likert scale in which 1, 2, 3, 4, 5, 6, and 7 corresponded to strongly disagree, disagree, slightly disagree, neither agree nor disagree, slightly agree, agree, and strongly agree, respectively.

#### Self-completed Occupational Performance lndex (SOPI) [27,28]

SOPI is used to measure social participation, and is based on the Canadian Model of Occupational Performance (CMOP). SOPI contains 9 items in 3 domains: productivity (3 items; score range, 3–15), leisure (3 items; score range, 3–15), and self-care (3 items; score range, 3–15). SOPI is evaluated using a 5 point scale from 1 (strongly disagree) to 5 (strongly agree) with the 3 scales summed to get the total score. Higher SOPI total score indicates higher social participation status.

#### Japanese version of 36-item short form health survey (SF-36) [29,30]

SF-36 is used to measure health-related quality of life and contains 36 items in 8 dimensions: physical functioning (10 items), role limitations due to physical health problems (4 items), bodily pain (2 items), social functioning (2 items), general mental health (5 items), role limitations due to emotional problems (3 items), energy/vitality (4 items), and general health perceptions (5 items). Moreover, SF-36 has developed algorithms to calculate 3 component summary measures: Physical Component Summary Scale Score (PCS), Mental Component Summary Scale Score (MCS), and Role/Social component summary Scale Score (RCS). This study utilized the 3 component summary measures of SF-36.

#### Japanese version of Occupational Self-Assessment (OSA) [31]

OSA is a self-reported measure that evaluates a patient’s own perceptions of their occupational competence and identity. OSA contains 21 items on 3 scales: performance capacity (11 items), habituation (one’s pattern of occupational engagement; 5 items), and volition (one’s motivation for participation; 5 items). OSA is measured with a 4 point response (i.e., 4 = I do this extremely well, 3 = I do this well, 2 = I have some difficulties doing this, and 1 = I have a lot of problems doing this).

### Statistical Analysis

SPSS Statistics and AMOS (http://www.spss.com) were used to analyze sample characteristics, construct validity, internal consistency reliability, structural validity, hypothesis testing (convergent and discriminant validity), concurrent validity, predictive validity, and test–retest reliability. Exametrika (http://antlers.rd.dnc.ac.jp/~shojima/exmk/index.htm) was used for the item responses. Mplus 7.3 (http://www.statmodel.com) was used for the multiple group analysis.

#### Sample characteristics

Participant demographics were summarized using descriptive analyses. The normal distribution of all scores of CAOD was examined using the Kolmogorov–Smirnov test (p < 0.05).

#### Construct validity

The factor structure of the 40 item version of the prototype CAOD was determined by performing exploratory factor analysis (EFA) using maximum likelihood estimation and promax rotation. Item reduction was performed using item floor effects or ceiling effects. Moreover, items not loading on a factor (factor loading of <0.4) or loading on more than 1 factor were eliminated from the scale. Analysis was then performed on the reduced item set. Percentage of variance accounted for by a factor was estimated using eigenvalues.

#### Structural validity

We performed an EFA followed by a confirmatory factor analysis (CFA) using maximum likelihood estimation. We utilized 2 indices to assess how well the model fits the data. The first index was the comparative fit index (CFI), with critical values of >0.95 [32]. The second index was the root mean square error of approximation (RMSEA). The critical values of RMSEA of 0.08–0.10 show a mediocre fit and those of <0.08 indicate a good fit [33].

#### Hypothesis testing (convergent and discriminant validity)

We performed the square of the correlation between the factors and the average variance extracted (AVE) for CAOD. To analyze discriminant validity, we compared the squared correlation between each pair of constructs against the average of AVE for the factor structure of CAOD. Convergent validity was checked to see whether the square root of each AVE value belonging to each latent construct was > 0.5.

#### Internal consistency reliability

Internal consistency reliability was evaluated using Cronbach’s alpha coefficient.

#### Concurrent validity

We assessed the concurrent validity of CAOD items by examining the relationships between SOPI and SF-36. We used the Spearman’s nonparametric correlation.

#### Predictive validity

We assessed the predictive validity of CAOD by examining its relationship to OSA. The assessment of the predictive validity of CAOD was performed by comparing the results of CAOD to OSA after a period of 1 week using Spearman’s correlation. If we obtain the correlation, there is likely to be occupational dysfunction in the future.

#### Test–retest reliability

The test–retest reliability of CAOD was evaluated by comparing baseline results to results at 1 week follow up using Spearman’s correlation.

#### Item response

The statistical models used in our analyses are based on the graded item response theory (IRT) using maximum likelihood estimation. IRT estimated item slope parameters and item difficulty parameters in CAOD. Moreover, IRT estimated CAOD’s total information curve (TIC) and item information curve (IIC), which indicates the occupational dysfunction level at which a response in a given category or higher becomes probable.

#### Robustness of CAOD

Robustness of CAOD was tested using a latent class model based multiple-group confirmatory factor analysis. We compare a four model: 1) configural invariance, 2) weak measurement invariance, 3) strong measurement invariance, and 4) structural invariance. To assess the model, three fit indices were used, including the Akaike’s Information Criterion (AIC), Bayesian Information Criterion (BIC), and sample-size adjusted BIC [34, 35].

## Results

### Sample Characteristics

In this study, 419 undergraduate students (57.3% response rate) responded, including 201 males, 210 females, and 8 unknowns, with an average age of 19.9 ± 2.09 years. Participants included 107 nursing students, 159 physical therapy students, 151 occupational therapy students, and 2 students from an unknown department. The Kolmogorov–Smirnov test showed that all scores had normal distribution.

### Construct validity

Table 1 shows the result of EFA for the 40-item prototype CAOD analysis. No floor and ceiling responses were observed. We determined the underlying factor structure of the item set. The 4 factors and 16 items were created from the EFA procedure and includes occupational marginalization (6 items), occupational imbalance (4 items), occupational alienation (3 items), and occupational deprivation (3 items). This factor structure was model fit to OBP2.0.

**Table 1:** Construct validity and internal consistency reliability of CAOD.

Items		Factor 1	Factor 2	Factor 3	Factor 4	Common factor
CAOD 16 Items α = 0.902						
**Factor 1 Occupational marginalization α = 0.826**						
Item14	It is thought that it is carried out by treatment different from the surrounding person.	**0.782**	−0.027	−0.026	0.102	0.609
Item29	I have opinions but nobody hears them.	**0.765**	−0.034	0.04	0.043	0.551
Item19	Criticism from a close friend is disheartening.	**0.696**	−0.016	−0.158	0.169	0.454
Item4	My hard work is not appreciated.	**0.683**	−0.037	0.062	0.009	0.464
Item37	I was concerned with a friend’s stress relief.	**0.605**	0.118	0.042	−0.152	0.469
Item38	It is talking with the partner who is not pleasant by force.	**0.566**	0.04	0.165	−0.125	0.469
**Factor 2 Occupational imbalance α = 0.874**						
Item21	My busy life has led to lack of sleep.	0.043	**0.868**	−0.103	−0.014	0.598
Item16	There is no time to rest, and I am tired.	0.014	**0.815**	0.052	0.043	0.664
Item11	Daily life is becoming very busy and increasingly exhausting.	−0.061	**0.798**	0.106	−0.015	0.639
Item1	I am so busy that the rhythm of my life is confused.	0.014	**0.73**	−0.052	0.044	0.521
**Factor 3 Occupational deprivation α = 0.837**						
Item13	I cannot enjoy my favorite activities.	−0.003	−0.071	**0.855**	0.081	0.578
Item12	There is no opportunity to carry out that what I consider important for its own sake.	0.052	0.117	**0.703**	−0.047	0.525
Item2	There is no place where I can enjoy hobbies.	0.028	−0.020	**0.696**	−0.004	0.434
**Factor 4 Occupational alienation α = 0.838**						
Item18	I feel that my life has no meaning.	0.069	0.030	−0.125	**0.809**	0.499
Item23	There is no sense of accomplishment in daily life.	−0.029	0.018	0.068	**0.793**	0.539
Item28	Daily life has become tedious.	−0.006	0.001	0.265	**0.630**	0.572
**Factor correlation**						
	Factor 1	1.000				
	Factor 2	0.372	1.000			
	Factor 3	0.641	0.235	1.000		
	Factor 4	0.566	0.509	0.588	1.000	

### Structural validity

**Fig 1:**
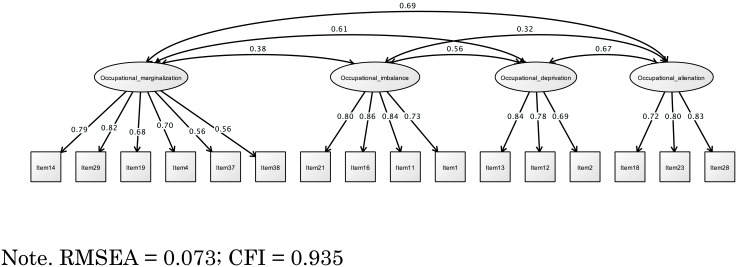
Structural validity of CAOD.

Fig 1 shows the results of CFA. On the basis of EFA, the CFA of CAOD was a good estimate of the model fit (RMSEA = 0.073; CFI = 0.935).

### Hypothesis testing (convergent and discriminant validity)

Table 2 shows the results of hypothesis testing. In brief, CAOD had convergent and discriminant validity.

**Table 2:** Hypothesis testing of CAOD.

CAOD	Convergent validity (AVE ≧ 0.5)	Discriminant validity (Factor correlation / 2 < AVE)
Factor 1	0.481 ≦ 0.5	0.142; 0.477 < 0.481
Factor 2	0.654 ≧ 0.5	0.101; 0.316 < 0.654
Factor 3	0.593 ≧ 0.5	0.316; 0.444 < 0.593
Factor 4	0.611 ≧ 0.5	0.101; 0.477 < 0.611

AVE = Average Variance Extracted

### Internal consistency reliability

Table 1 shows the result related to internal consistency. The internal consistency of CAOD (total score and all subscales) was within the acceptable range, between 0.826 and 0.902.

### Concurrent validity

Table 3 shows the result related to concurrent validity. The concurrent validity was confirmed by the correlations between the 16 items of CAOD and SOPI and SF-36. CAOD showed a negative correlation to SOPI total score (r = 0.097 to −0.331, p < 0.01). Moreover, CAOD showed a negative correlation to the MCS and RCS components of SF-36 (r = −0.133 to −0.421, p < 0.01).

**Table 3:** Concurrent validity of CAOD.

		SF-36		
	SOPI total score	PCS	MCS	RCS
Item1	−0.168**	−0.071	−0.276**	−0.172**
Item2	−0.318**	0.063	−0.369**	−0.244**
Item4	−0.159**	−0.006	−0.252**	−0.202**
Item11	0.097**	−0.061	−0.286**	−0.158**
Item12	−0.224**	0.024	−0.276**	−0.239**
Item13	−0.286**	0.096	−0.335**	−0.268**
Item14	−0.250**	0.007	−0.329**	−0.207**
Item16	−0.245**	−0.041	−0.322**	−0.202**
Item18	−0.252**	0.002	−0.297**	−0.140**
Item19	−0.191**	−0.025	−0.268**	−0.160**
Item21	−0.167**	−0.069	−0.228**	−0.156**
Item23	−0.331**	−0.022	−0.421**	−0.163**
Item28	−0.266**	−0.018	−0.421**	−0.133**
Item29	−0.216**	0.052	−0.363**	−0.212**
Item37	−0.130**	−0.015	−0.209**	−0.216**
Item38	−0.102**	0.009	−0.247**	−0.196**

PCS = Physical component summary, MCS = Mental component summary, RCS = Role / Social component summary,

** = Significant at 1% level.

### Predictive validity

Table 4 shows the result related to predictive validity. The predictive validity was confirmed by the correlations between the 16 items of CAOD and OSA. CAOD showed a negative correlation to OSA (r = −0.116 to −0.425, p < 0.01).

**Table 4:** Predictive validity and test–retest reliability of CAOD.

	Predictive validity		Test-retest reliability
	OSA occupational competence	OSA occupational identity	
Item1	−0.244**	−0.116**	0.619**
Item2	−0.305**	−0.187**	0.659**
Item4	−0.306**	−0.222**	0.578**
Item11	−0.233**	−0.077	0.625**
Item12	−0.237**	−0.074	0.516**
Item13	−0.356**	−0.186**	0.622**
Item14	−0.363**	−0.147*	0.647**
Item16	−0.282**	−0.109	0.640**
Item18	−0.343**	−0.129*	0.635**
Item19	−0.289**	−0.176**	0.639**
Item21	−0.228**	−0.136*	0.609**
Item23	−0.425**	−0.184**	0.637**
Item28	−0.360**	−0.245**	0.678**
Item29	−0.403**	−0.168**	0.600**
Item37	−0.243**	−0.210**	0.552**
Item38	−0.233**	−0.206**	0.580**

* = Significant at 5% level

** = Significant at 1% level

### Test–retest reliability

Table 4 shows the results related to test–retest reliability. CAOD displayed strong test–retest reliability (r = 0.516 to 0.678, p < 0.01).

### Item response

Table 5 shows the results of item slope parameters (α) and item difficulty parameters (β). Overall, 16 items on CAOD demonstrated satisfactory item response, with item slopes ranging from 1.091 to 1.393. Item difficulty parameters ranged from −CAOD demonstrated satisfa response of CAOD provided the appropriate discrimination index and difficulty index.

**Table 5:** Item response of CAOD.

Item	α	β1	β2	β3	β4	β5	β6
Item1	1.091	−1.829	−1.288	−0.825	−0.38	0.577	1.275
Item2	1.251	−1.149	−0.591	−0.009	0.413	1.071	1.713
Item4	1.274	−1.126	−0.5	0.039	1.039	1.687	2.589
Item11	1.183	−1.935	−1.477	−0.922	−0.323	0.473	1.331
Item12	1.343	−1.459	−0.688	−0.106	0.556	1.331	1.829
Item13	1.393	−0.989	−0.407	0.235	0.75	1.331	1.797
Item14	1.363	−0.816	−0.31	0.222	1.019	1.663	2.069
Item16	1.307	−1.235	−0.695	−0.228	0.278	0.94	1.533
Item18	1.169	−1.103	−0.695	−0.342	0.106	0.808	1.573
Item19	1.163	−0.658	−0.051	0.387	1.029	1.616	2.069
Item21	1.134	−1.126	−0.68	−0.142	0.304	1.008	1.459
Item23	1.301	−1.248	−0.711	−0.088	0.38	0.989	1.533
Item28	1.352	−1.081	−0.57	0.033	0.535	1.209	1.713
Item29	1.325	−1.007	−0.301	0.352	1.102	1.827	2.588
Item37	1.135	−0.547	0	0.498	1.136	1.661	2.122
Item38	1.14	−0.658	−0.185	0.272	0.825	1.346	1.862
Average	1.245	- 1.122	- 0.571	- 0.039	0.548	1.221	1.815
Standard deviation	0.099	0.382	0.394	0.401	0.480	0.409	0.393

α = Item slope parameter

β = Item difficulty parameter

**Fig 2:**
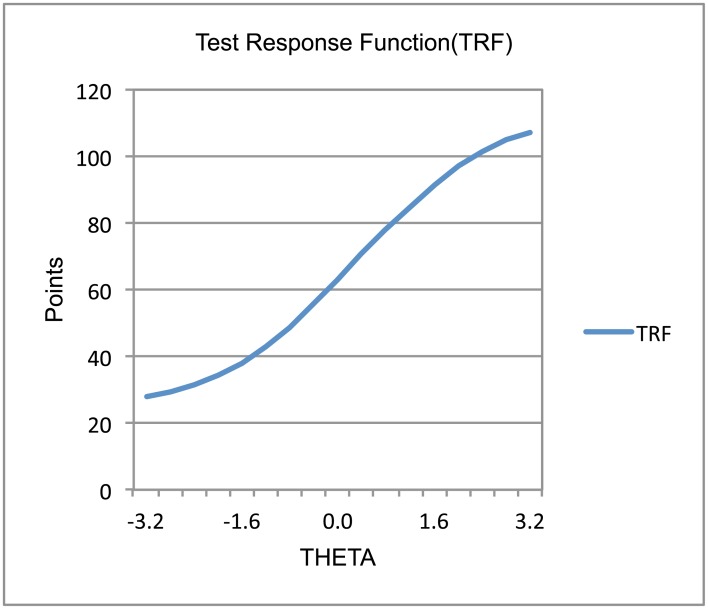
Test response function of CAOD.

**Fig 3:**
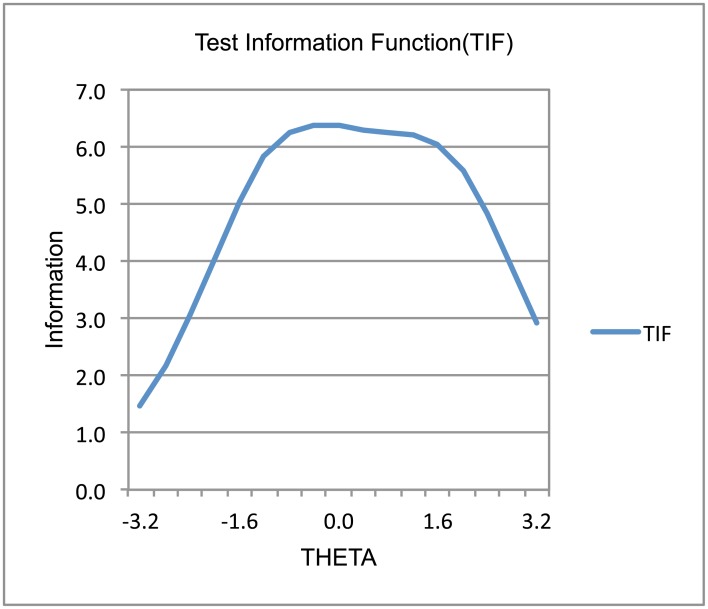
Test information function of CAOD.

Figs 2 and 3 present the test response function (TRF) and test information function (TIF) related to CAOD. Overall, CAOD measured an approximately equally wide range of occupational dysfunctions with high precision. Moreover, CAOD was slightly more precise at low levels of occupational dysfunction (i.e., higher theta values).

### Robustness of CAOD

Table 6 shows the result related to robustness of CAOD. CAOD displayed the weak measurement invariance of the gender groups (AIC = 20779.419, BIC = 21269.688, sample-size adjusted BIC = 20882.557) and department groups (AIC = 21379.654, BIC = 21843.183, sample-size adjusted BIC = 21478.259).

**Table 6:** Robustness of CAOD.

Gender	AIC	BIC	Sample-size BIC
Configural invariance	20861.241	21403.751	20975.369
Weak measurement invariance	**20779.419**	**21269.688**	**20882.557**
Strong measurement invariance	20847.827	21289.873	20940.821
Structural invariance	20874.311	21352.523	20974.913
Department	AIC	BIC	Sample-size BIC
Configural invariance	21524.493	22137.157	21654.822
Weak measurement invariance	**21379.654**	**21843.183**	**21478.259**
Strong measurement invariance	21449.068	21864.228	21537.383
Structural invariance	21516.942	22000.624	21619.883

Note: We have adopted a Weak measurement invariance that is shown in boldface.

## Discussion

### Psychometric properties of CAOD

We developed and validated the Classification and Assessment of Occupational Dysfunction (CAOD) as a new, self-administered measure for evaluating occupational dysfunction in undergraduate students. Several of our results suggest evidence for the validity and reliability of CAOD (Tables 1 to 5, Figs 1 to 3). To the best of our knowledge, this is the first study on the development of an assessment for classification of occupational dysfunction.

Overall, CAOD had a good model fit. The construct and structural validity of CAOD were assessed by EFA and CFA, and they indicated a good model fit (Fig 1 and Table 1). The hypothesis testing of this study demonstrated a good value for convergent and discriminant validity of CAOD (Tables 3 and 4). In addition, occupational marginalization may need to be reexamined in the future because we obtained a rather small value. Assessed by Cronbach’s alpha coefficient, internal consistency was acceptable (Table 1). In summary, these facts clearly prove that the model of OBP2.0 fits the data. That is, the final version of CAOD was formed by a concept similar to the classification of occupational dysfunction in OBP2.0. Therefore, we think that CAOD is empirically and theoretically well supported.

Several results suggest evidence for the concurrent and predictive validity of the measure. First, concurrent validity was assessed by comparison of CAOD, SOPI, and SF-36 (Table 3). A modest negative correlation between CAOD, SOPI, and SF-36 (MCS and RCS) was observed. Occupational dysfunction is identified as a major health-related problem. The results of this study demonstrate that occupational dysfunction is related to barriers to social participation as well as to decline in health-related quality of life. In addition, it was found that occupational dysfunction and SF-36 PCS (includes physical functioning, body pain, and role physical) correlated poorly. This suggests that CAOD and SF-36 PCS each represent a different aspect of the subjective experience. Second, predictive validity assessed by comparison of CAOD and OSA (Table 4) demonstrates a modest correlation between CAOD and OSA. The results regarding the predictive validity of CAOD showed a moderate negative value for occupational competency and occupational identity.

Test–retest reliability of CAOD was very good, with high correlations (Table 4). Although occupational dysfunction may change over time, the reproducibility of CAOD in this study was high.

IRT was used to assess individual item characteristics of CAOD (Table 5). CAOD has high item slope parameters, in the range of 1.091–1.393. Item difficulty parameter scores for CAOD are very wide, ranging from −0.547 to 2.589. Moreover, the results indicate that TRF and TIF of CAOD were sufficiently identified (Figs 2 and 3). The amount of information for CAOD has been sufficiently identified. These results clearly demonstrate strong support for good item response of CAOD.

Results from the latent class model based multiple-group CFA indicate that the CAOD is a structurally valid four factors structure measure of occupational dysfunction in both gender groups and department groups. Overall, CAOD was able to show the robustness of the results.

### Practical implications

There are several practical implications for developing CAOD. A valid and reliable assessment is needed for evaluating and intervening with preventive occupational therapy programs based on OBP2.0. CAOD is a screening tool intended to collect broad range information about a person’s occupational dysfunction. Moreover, CAOD can be used as a tool to facilitate therapy of occupational dysfunction that includes occupational imbalance, occupational deprivation, occupational alienation, and occupational marginalization. CAOD may assist the patient and the occupational therapist in establishing goals and plans of care for addressing occupational dysfunction. We can prepare CAOD for undergraduate students. Undergraduate students have many lifestyle problems. In the future, we can understand the relationship of occupational dysfunction and mental illness. In addition, we can use CAOD for other participants in preventive occupational therapy.

### Limitations

This study design has limitations. First, the survey was conducted with only undergraduate students, raising the question of the generalizability. Moreover, the psychometric properties of CAOD in samples with medical diseases are unknown and should be examined in future studies. Despite these limitations, CAOD is a potentially useful tool for estimating and monitoring the classification of occupational dysfunction.

## Conclusion

Overall, the study findings suggest that CAOD is a valid, reliable assessment for assessing occupational dysfunction in undergraduate students. CAOD demonstrates valid psychometric properties for measuring occupational dysfunction, and can be utilized for preventive occupational therapy.
